# Validity and reliability of the Questionnaire for Compliance with Standard Precaution

**DOI:** 10.1590/S0034-8910.2015049005975

**Published:** 2015-12-16

**Authors:** Marília Duarte Valim, Maria Helena Palucci Marziale, Miyeko Hayashida, Fernanda Ludmilla Rossi Rocha, Jair Lício Ferreira Santos

**Affiliations:** I Departamento de Enfermagem. Centro Universitário da Fundação de Ensino Octávio Bastos. São João da Boa Vista, SP, Brasil; II Departamento de Enfermagem Fundamental. Escola de Enfermagem de Ribeirão Preto. Universidade de São Paulo. Ribeirão Preto, SP, Brasil; III Departamento de Enfermagem. Seção de Apoio Laboratorial. Escola de Enfermagem de Ribeirão Preto. Universidade de São Paulo. Ribeirão Preto, SP, Brasil; IV Departamento de Medicina Social. Faculdade de Medicina de Ribeirão Preto. Universidade de São Paulo. Ribeirão Preto, SP, Brasil

**Keywords:** Nurses, Universal Precautions, Training, Questionnaires, Translations, Reproducibility of Results, Validity of Tests, Validation Studies

## Abstract

**OBJECTIVE:**

: To evaluate the validity and reliability of the Questionnaire for Compliance with Standard Precaution for nurses.

**METHODS:**

: This methodological study was conducted with 121 nurses from health care facilities in Sao Paulo’s countryside, who were represented by two high-complexity and by three average-complexity health care facilities. Internal consistency was calculated using Cronbach’s alpha and stability was calculated by the intraclass correlation coefficient, through test-retest. Convergent, discriminant, and known-groups construct validity techniques were conducted.

**RESULTS:**

: The questionnaire was found to be reliable (Cronbach’s alpha: 0.80; intraclass correlation coefficient: (0.97) In regards to the convergent and discriminant construct validity, strong correlation was found between compliance to standard precautions, the perception of a safe environment, and the smaller perception of obstacles to follow such precautions (r = 0.614 and r = 0.537, respectively). The nurses who were trained on the standard precautions and worked on the health care facilities of higher complexity were shown to comply more (p = 0.028 and p = 0.006, respectively).

**CONCLUSIONS:**

: The Brazilian version of the Questionnaire for Compliance with Standard Precaution was shown to be valid and reliable. Further investigation must be conducted with nurse samples that are more representative of the Brazilian reality. The use of the questionnaire may support the creation of educational measures considering the possible gaps that can be identified, focusing on the workers’ health and on the patients’ safety.

## INTRODUCTION

The standard precautions include hand hygiene, use of personal protective equipment (PPE), prevention of accidents with sharp objects, environment cleaning and disinfection measures, cough etiquette, and safe practices during injections.^23^ When correctly implemented, they are the main strategy to protect workers against exposure to biological materials and to prevent health care-related infections.^6,17^

Even with the creation and disclosure of protocols regarding standard precautions in health care institutions, workplace accidents with potentially contaminated biological material are frequent.^5,16^ Health care-related infections are the most common and studied adverse effect from hospitalization. They are the main health care problem in developed and developing countries due to deaths, prolonged hospitalization periods, and negative effects to patients to the well-being of the nation.^29^

Compliance to standard precautions is below recommended levels worldwide.^3,7,26^ Some variables are pointed out to influence compliance to standard precautions, such as: previous training on standard precautions;^2,14^ perceived organizational safety;^4^ perceived obstacles to comply with standard precautions;^11^ perceived self-efficacy;^14^ complexity of health care facilities – once compliance was shown to be higher in larger, more complex facilities^12,14^ –, and knowledge regarding such measures.^19^

Compliance to standard precaution measures as a primary strategy to prevent workplace accidents and professional diseases resulting from contact with potentially-contaminated biological material is essential and must be evaluated by health care institutions, considering individual variables and variables from the very work organization.

Despite the need for using valid and reliable instruments to evaluate constructs of interest,^28^ few instruments in the literature aim to measure such construct.^2,8,14,19,27^ The Brazilian Portuguese version^14^ of the Questionnaire for Compliance with Standard Precaution^27^ has adequate psychometric characteristics and covers important concepts regarding compliance with standard precaution measures, such as hand hygiene, safe practices while handling sharp objects, and use of PPE – safety gloves, masks, glasses, and aprons.

The Questionnaire for Compliance with Standard Precaution, which was created and validated in a sample of 1,444 Chinese nurses,^14^ has proper psychometric characteristics. It comprises twenty Likert scale questions with scores from 0 to 4 points, with score ranges from zero to eighty points. Four points are added with every “always” answer, whereas “never” answers add no points whatsoever. The only inverse item is the number 20 one. The related questionnaire was created based on the Questionnaire of Practice of Standard Precaution that was developed by Askarian et al^2^ and on the international guidelines^23^ on standard precaution measures.

The aim is to resume the adaptation process of the Questionnaire for Compliance with Standard Precaution (QCSP),^27^ by the analysis of measurement equivalence. Three main approaches are suggested: evaluation of the dimensional structure of the instrument, including the adaptation of items it comprises; analysis of the reliability of information; and the verification of the instrument validity.^21^

As a theoretical and methodological reference, the process for validating measurement instruments proposed by Pasquali was adopted.^20^ According to that author, instruments are only validated when a previous theory serves as base for the behavioral representation, which constitutes a hypothesis that can be deduced from that theory.

The aim of this study was to evaluate the validity and reliability of the Questionnaire for Compliance with Standard Precaution that was adapted for nurses.

## METHODS

This was a methodological study, and it was conducted with nurses from health care institutions in Sao Paulo’s countryside, Brazil. A sample of nurses belonging to high-complexity facilities was randomly picked from one of the municipalities in Sao Paulo countryside, and so was another one with nurses from medium-complexity health care facilities from another municipality in the countryside of the same state. A criterion to choose different institutions according to their complexity levels was adopted to enable the conduction of known or contrasted groups construct validity.

The high-complexity health care facilities were represented by a teaching hospital and by an emergency unit tied to such hospital, which were classified as being of size IV.^18^ The medium-complexity health care facilities were represented by a private charity hospital, a private hospital, and an emergency unit that belonged to a private health care service.

According to Sapnas and Zeller,^22^ samples of at least 50 and maximum 100 subjects are sufficient when one intends to evaluate the psychometric properties of instruments for measuring constructs. The authors believe that 10 respondents per item represents sample size overkill.

The number of nurses who were connected with the emergency unit (125) and to the teaching hospital (286) in 2011 totaled 411 nurses, according to information obtained by the human resources department. The number of nurses working in the three medium-complexity health care facilities was 39. According to Sapnas and Zeller^22^ and to possible losses and refusals, a sample of 120 nurses was randomly picked by Statistical Package for Social Science (SPSS) software, version 16.0, who worked at the high-complexity teaching hospital. The 39 nurses from the medium-complexity services were chosen to be included.

Nurses included could not be on vacation, medical or maternity leave, or on any other kinds of leave. They were also required to have over six months’ professional experience. The ones who performed clerical duties exclusively or the ones who were being given training when the data were collected were excluded.

The self-administered questionnaires were left in a sealed box in the nursing management offices of each department for a week, so they could later be collected. That procedure ensures increased anonymity to subjects, which results in data that better represent reality. In the departments where it was not possible to leave the collection box, the nurses were asked to stay with the questionnaires so they could later be collected by the researcher, who did so in the workers’ next shift, in three consecutive attempts.

The data were collected from September to December 2012. A sociodemographic questionnaire, a Safety Perception Scale, and a Scale of Perceived Obstacles to comply with standard precautions were used in versions adapted and validated for Brazil,^4^ as well as the QCSP mentioned.^27^

A hypothesis for convergent (positive) correlation was established between the total measure of QCSP and the perceived organizational safety measure for the convergent construct validity. For discriminant construct validity, a hypothesis was established for negative correlation between the total measure of QCSP and the increased perception of obstacles to follow them.

The Likert Safety Perception Scale was found to have Cronbach’s alphas of 0.80 for item “safety-supporting management actions” and 0.69 for item “feedback from safe practices”. The Scale of Perceived Obstacles to comply with standard precautions, also of the Likert type, was found to have a Cronbach’s alpha of 0.69. The scales are part of a model to explain compliance with standard precautions, which comprises individual, organizational, and work-related dimensions.^4^

A hypothesis was established that nurses who received training on the standard precautions complied more with those measures than the ones who reported not having received training, for the verification of known-groups validity. The known-groups validity of QCSP tested the hypothesis that nurses working at high-complexity units complied more with standard precautions than the ones who worked in medium-complexity units.

The content validity was tested by the presence of maximum and minimum effects. Reliability was tested using test-retest, which represents the ability of a test to measure the same subjects at different times and to produce identical results; i.e., the correlation between the two measures that were obtained at different times must be the closest to 1 as possible.^20^ To perform the retest, a sample of 30 nurses was selected, with workers from high-complexity health care facilities , according to the interpolation of values as indicated by the literature,^9^ whose results point towards a sample of 28 subjects.

The convergent and discriminant construct validity was conducted using Pearson’s correlation with the Safety Perception Scale and the Scale of Perceived Obstacles to comply with standard precautions. Values below 0.30 correspond to weak correlations with low clinical applicability; values between 0.30 and 0.50 are considered moderate correlations; and the ones above 0.50, strong correlations.^1^ For the known-groups validity analysis, student’s t test was used. The tests were applied after verification of normality by the Shapiro-Wilk test.

Internal consistency was tested by the calculation of Cronbach’s alpha, whose values between 0.70 and 0.90 were considered to be acceptable.^10^ The intraclass correlation coefficient (ICC) was calculated for stability analysis, by comparing the score obtained after application of the questionnaire in the test-retest. To apply the retest, a period from seven to 14 days was allowed to elapse between measurements, as recommended by the literature. It had a 95% confidence interval (95%CI).^25^

An ICC of at least 0.65 was attempted to be measured, with a 0.3 range in the confidence interval and a 5% significance level. The value of 0.65 for ICC followed the recommendation of Landis and Koch^13^ for substantial agreement.

A significance level of 0,05 was adopted for all hypothesis tests. The tests comprised the significance or lack thereof or correlations, using the usual Pearson’s correlation technique and the repeated-measures ANOVA for ICC.

The data were processed and analyzed by SPSS, version 16.0, for Windows 7.0. The data were entered twice to be compiled, aiming to minimize potential typing errors. The study was approved by the Research Ethics Committee of Escola de Enfermagem de Ribeirão Preto of the Universidade de São Paulo (Protocol 1,306/2011). The standards involving research on human beings from Resolution 196/96 of Brazil’s National Health Council were followed. All subjects signed consent forms.

After the losses and refusals were calculated, the final sample comprised 121 nurses, 91 of whom from high-complexity units and 30 of them from medium-complexity units. Response percentages were 75.8% for high-complexity units and 77.0% for the remaining health care facilities. Regarding the subjects included for participation in the retest, two subjects among the 30 selected refused to take part. The final sample comprised 28 subjects.

## RESULTS

The vast majority (90.9%) of the professionals were female workers. Among the nurses from high-complexity units, 48.8% of them worked at the teaching hospital and 26.4% at the emergency unit connected to that hospital. A total of 13.2% of professionals in medium-complexity units worked at the charity Santa Casa and 11.6% of them worked at the private hospital and at the emergency unit.

The average age of workers was 36.2 years (SD = 8.95), median of 34, maximum age of 58 and minimum age of 23 years. The average length of experience was 10.16 years (SD = 7.22), median of eight years. The longest professional experience period was 30 years in the profession. The minimum one was three months.

In the descriptive analysis of QCSP, an average compliance of 62.2 points was obtained (SD = 8.47) with a median of 63.0. The minimum score obtained was 34 and the maximum one was 78 points (Table 1).

**Table 1:** Descriptive statistics of the values that were obtained by the questionnaire for compliance with the standard precaution measures by nurses (n = 121). Health care services in municipalities in the countryside of Sao Paulo, Southeastern Brazil, 2012.

Variable	Always (4)		Often (3)		Sometimes (2)		Seldom (1)		Never (0)	
	f	%	f	%	f	%	f	%	f	%
1. Sanitizes hands in between treating different patients.	73	60.3	45	37.2	3	2.5	–	–	–	–
2. Sanitizes hands after taking off gloves.	89	73.5	29	24.0	3	2.5	–	–	–	–
3. Sanitizes hands immediately after touching potentially-contaminated biological materials.	111	91.7	10	8.3	–	–	–	–	–	–
Reported frequency of wearing gloves in procedures in which there are possibilities for getting in contact with the potentially-contaminated biological materials listed below.										
4. Blood collection.	63	52.1	33	27.3	17	14.0	8	6.6	–	–
5. Procedures involving the possibility of touching urine or feces.	96	79.3	20	16.5	3	2.5	2	1.7	–	–
6. Procedures involving the possibility of touching a patient’s non-intact skin.	89	73.6	20	16.5	9	7.4	3	2.5	–	–
7. Procedures involving the possibility of touching a patient’s mucous membrane.	91	75.2	22	18.2	5	4.1	3	2.5	–	–
8. Procedures involving the possibility of touching a patient’s airway discharges.	109	90.0	7	5.8	2	1.7	2	1.7	1	0.8
9. Intramuscular or subcutaneous injections.	16	13.2	38	31.4	31	26.5	22	18.2	14	11.6
10. Dressing of wounds.	83	68.6	21	17.4	16	13.2	1	0.8	–	–
11. Cleaning for blood removal.	105	86.8	11	9.1	5	4.1	–	–	–	–
12. Venipunctures.	50	41.3	34	28.1	30	24.8	7	5.8	–	–
13. Contact with blood samples.	48	39.7	47	38.8	14	11.6	9	7.4	3	2.5
14. Wears a protection mask when there is a possibility of touching drops of blood, bodily fluids, discharges, or dejecta.	20	16.5	69	57.0	23	19.0	7	5.8	2	1.7
15. Wears protection glasses when there is a possibility of touching drops of blood, bodily fluids, discharges, or dejecta.	15	12.4	44	36.4	43	35.5	16	13.2	3	2.5
16. Wears a protection apron when there is a possibility of touching drops of blood, bodily fluids, discharges, or dejecta.	15	12.4	35	28.9	24	19.8	30	27.9	17	14.0
17. Wears disposable caps and shoe covers when there is a possibility of touching drops of blood, bodily fluids, discharges, or dejecta.	9	7.5	17	14.0	26	21.5	30	24.8	39	32.2
18. Does not recap used needles or uses the one-hand recapping method.	53	43.8	23	19.0	27	22.3	10	8.3	8	6.6
19. Disposes needles, blades, and other sharp materials in containers that are specific for that purpose.	119	98.3	2	1.7	–	–	–	–	–	–
20. After workplace accidents with potentially-contaminated sharp materials, immediately squeezes the affected part, disinfects it, and dresses the wound.	41	33.8	11	9.1	5	4.1	10	8.3	48	36.7

Although 91.7% of the nurses have reported they always washed their hands after getting in contact with potentially-contaminated biological material, 39.7% of total nurses in the sample did not clean them while treating different patients. Around 52.1% of the nurses always used gloves when collecting blood, 13.2% used them when giving patients intramuscular or subcutaneous injections, 68.6% while dressing wounds, and 41.3% during venipunctures. Regarding protection masks, 16.5% of the nurses wore them when there was a possibility for getting in contact with potentially-contaminated biological material; 35.5% of them sometimes used protection glasses, and 15.7% seldom or never wore them. A total of 19.8% of nurses seldom or never wore protection aprons.

Only items 9, 15, and 16 suffered no influence from maximum and minimum effects (Table 1). The existence of maximum and minimum effects was observed when over 15.0% of the subjects opted for the lowest or for the highest possible score in the response scale, respectively.

The total Cronbach’s alpha coefficient was 0.80 (Table 2).

**Table 2:** Item-total correlation coefficient, total alpha value of the 20 items from the questionnaire for compliance with standard precaution, and alpha values when each of the items was excluded. Health care services in municipalities in the countryside of Sao Paulo, Southeastern Brazil, 2012.

Questionnaire for Compliance with Standard Precaution	Average, if removed item	Variance, if removed item	Corrected item-total correlation	Cronbach’s alpha, if removed item
1. Sanitizes hands in between treating different patients.	78.05	65.62	0.42	0.78
2. Sanitizes hands after taking off gloves.	77.91	68.16	0.14	0.79
3. Sanitizes hands immediately after touching potentially-contaminated biological materials.	77.71	68.52	0.25	0.79
Reported frequency of wearing gloves in procedures in which there are possibilities for getting in contact with the potentially-contaminated biological materials listed below.				
4. Performs blood collection.	78.39	59.73	0.63	0.76
5. Executes procedures involving the possibility of touching urine or feces.	77.90	64.32	0.52	0.78
6. Executes procedures involving the possibility of touching a patient’s non-intact skin.	78.02	63.94	0.46	0.78
7. Executes procedures involving the possibility of touching a patient’s mucous membrane.	77.98	63.64	0.50	0.78
8. Executes procedures involving the possibility of touching a patient’s airway discharges.	77.81	64.36	0.49	0.78
9. Gives intramuscular or subcutaneous injections.	79.49	57.35	0.59	0.76
10. Dresses wounds.	78.11	65.54	0.28	0.79
11. Cleans for blood removal.	77.81	65.68	0.48	0.78
12. Executes venipunctures.	78.61	58.97	0.68	0.76
13. Gets in contact with blood samples.	78.58	60.22	0.53	0.77
14. Wears a protection mask when there is a possibility of touching drops of blood, bodily fluids, discharges, or dejecta.	78.81	63.19	0.44	0.78
15. Wears protection glasses when there is a possibility of touching drops of blood, bodily fluids, discharges, or dejecta.	79.22	59.97	0.58	0.77
16. Wears a protection apron when there is a possibility of touching drops of blood, bodily fluids, discharges, or dejecta.	79.61	59.31	0.45	0.78
17. Wears disposable caps and shoe covers when there is a possibility of touching drops of blood, bodily fluids, discharges, or dejecta.	80.22	61.20	0.34	0.78
18. Does not recap used needles or uses the one-hand recapping method.	78.82	65.23	0.13	0.80
19. Disposes needles, blades, and other sharp materials in containers that are specific for that purpose.	77.65	69.38	0.16	0.79
20. After workplace accidents with potentially-contaminated sharp materials, immediately squeezes the affected part, washes it, disinfects it, and dresses the wound.	79.53	66.39	-0.001	0.83

The ICC obtained by the test-retest of the QCSP was 0.973 (95%CI 0.929;0.987), which means an almost perfect classification.

In regards to QCSP validity, strong correlation was found (r = 0.614 and p ≤ 0.001) between compliance to standard precautions and the higher perceived safety by the nurses. In regards to the convergent construct validity, strong correlation (r = -0.537 and p ≤ 0.001) was found between compliance to standard precautions and the smaller perception of obstacles to follow such precautions.

Among all the nurses included in this sample, 15 reported not having received training on standard precaution measures. The group of nurses who received training was found to have an average score of 66.8 (n = 15) in the QCSP, and the group that was not given training had an average of 62.8 (n = 98), with statistically significant differences (p = 0.028).

Compliance with standard precautions differed significantly among the groups (p = 0.006). The group of nurses from high-complexity health care facilities (n = 87) obtained an average compliance of 68, and the group from the remaining health care institutions (n = 28) obtained 63.2.

## DISCUSSION

When a certain construct is proposed to be measured, the instruments must be valid and reliable. The viability of a reliable instrument to measure compliance of nurses with standard precautions is important for the Brazilian context of occupational health care and for the safety of patients.

The descriptive analysis of the instrument showed the presence of maximum and minimum effects in the instrument. Only items 9, 15, and 16 suffered no influence from those effects. That may lead to negative repercussion regarding the validity of the instrument contents, which may indicate sub-optimal representativity of the construct investigated by the instrument items.^28^ A possible justification for the presence of a maximum effect is that studies observe self-reported compliance with indices above the ones that are really found by observational studies,^3,15^ as professionals tend to overestimate what they really execute in practice.

Regarding reliability, desirable internal consistency was observed – it is described as the ability to find correlation (homogeneity) between the items of an scale-fashioned instrument; that is, to find whether they measure the same theoretical construct they are proposed to. The measure of internal consistency obtained by Cronbach’s alpha is important and desirable when working with instruments aiming to measure a single construct through multiple items. Such value must range from 0.70 to 0.95.^10^

Also regarding internal consistency, items 2, 18, and 20 were found to have low correlation with the dimensions they belonged to, which may suggest dimensions that are different from the one verified in the instrument. However, none of those items, if removed, would change the alpha value by more than 5.0%, and that is why they were chosen to be kept until future studies indicate otherwise. Item 20 was the only one that was not answered by six nurses. If excluded, it would lead to a total alpha of 0.83. The item was kept because of iwts relevance. Verification using principal components was not conducted for the possible structural dimensions of the instrument. The high value of its internal consistency indicates its unidimensionality or, at least, the predominance of one dimension.

Stability was found to be satisfactory, which confirms that the tool can be applied at different times to detect possible changes in the studied sample.

Construct validity aims to support the instrument ability to measure what it intends to. A gap in that kind of validity can negatively affect the results obtained by the application of an instrument.^28^ The strong correlation that was found by the validity tests between compliance with standard precautions and the higher perceived organizational safety corroborates scientific findings.^4,11^ That reinforces the hypothesis that the instrument really measures the construct it is intended to. However, the correlation values undergo slightly different classification according to some studies,^24^ in which values between 0.4 and 0.6 are moderate correlations and values above 0.7 are considered to be strong ones.

Health care institutions with strong safety cultures have lower cases of workplace accidents as compared to institutions with weaker safety cultures. The smaller frequency of accidents is not only due to the presence of effective, well-developed safety programs in the institutions, but also to the management of those institutions through those programs, which indicates signs of commitment to the safety of their workers.^7^

Known-groups validity of QCSP strengthens that questionnaire. Permanent training and education of health care professionals are variables that increase compliance with safety measures, as they are essential measures to ensure policies for preventing and controlling health care-related infections and occupational safety measures are understood and adhered to.^3^

Known-groups validity was also shown to be satisfactory regarding the fact that nurses from high-complexity health care facilities comply more with standard precautions than the ones who work in medium-complexity health care facilities. Human and financial resources significantly vary among the types of health care facilities. Thus, teaching hospitals and institutions of higher complexity tend to enforce more efficient practices to control infection than municipal or charity hospitals, which are less complex and receive less financial incentives.^12^

The findings in this study confirm the QCSP is adapted and can be used for nurses in Brazil. Future findings can strengthen the instrument or show limitations in its use.

Regarding participation in training sessions by nurses, future research may investigate the topics studied, the methods used, and the periodicity of training courses conducted. Thus, it would be possible to have a more well-grounded descriptive analysis regarding the training offered. That may support the verification of variables that can possibly influence the compliance of the nurses with the standard precautions, besides cooperating with questionnaire validity and to support intervention proposals.

The study sample is not very representative of the reality of Brazilian nurses. Other studies are required, with a higher number of subjects and more heterogeneous, less localized samples. Investigations with samples of nurses working at several health care institutions from different regions in the country are recommended to evaluate regional interferences. The regionalism and the large territorial extension of Brazil, as well as the social, economic, and cultural characteristics that are peculiar to health care institutions must be considered.

The questionnaire for compliance with standard precautions had satisfactory reliability, which was calculated through internal consistency and stability. The related questionnaire was found to perform well in the tests for discriminant, convergent, and distinct-group construct validity. Future investigations shall resume the process to verify the measurement equivalence of the questionnaire by analyzing the dimensional validity of the main components of the instrument.

## References

[1] Ajzen I, Fishbein M (1980). Understanding attitudes and predicting social behavior.

[2] Askarian M, Mclaws M-L, Meylan M (2007). Knowledge, attitude, and practices related to standard precautions of surgeons and physicians in university-affiliated hospitals of Shiraz, Iran. Int J Infect Dis.

[3] Bledsoe BE, Sweeney RJ, Berkeley RP, Cole KT, Forred WJ, Johnson LD (2014). EMS provider compliance with infection control recommendations is suboptimal. Prehosp Emerg Care.

[4] Ajzen I, Fishbein M (1980). Understanding attitudes and predicting social behavior.

[5] Askarian M, Mclaws M-L, Meylan M (2007). Knowledge, attitude, and practices related to standard precautions of surgeons and physicians in university-affiliated hospitals of Shiraz, Iran. Int J Infect Dis.

[6] Bledsoe BE, Sweeney RJ, Berkeley RP, Cole KT, Forred WJ, Johnson LD (2014). EMS provider compliance with infection control recommendations is suboptimal. Prehosp Emerg Care.

[7] Brevidelli MM, Cianciarullo TI (2009). Fatores psicossociais e organizacionais na adesão às precauções-padrão. Rev Saude Publica.

[8] Centers for Disease Control and Prevention (CDC), Department of Health and Human Services (2010). Surveillance of occupationally acquired HIV/AIDS in healthcare personnel, as of December 2010.

[9] Centers for Disease Control and Prevention (CDC), National Center for Emerging and Zoonotic Infectious Diseases (2011). Infection prevention checklist for outpatient settings: minimum expectations for safe care.

[10] Centers for Disease Control and Prevention (CDC) (2008). Workbook for designing, implementing and evaluating a sharp injury prevention program.

[11] Chan R, Molassiotis A, Chan E, Chan V, Ho B, Lai CY (2002). Nurses’ knowledge of and compliance with universal precautions in an acute care hospital. Int J Nurs Stud.

[12] Doros G, Lee R (2010). Design based on intra-class correlation coefficients. Am J Biostat.

[13] Fayers PM, Machin D (2007). Quality of life: assessment, analysis and interpretation.

[14] Felix AMS, Victor ES, Malaguti SET, Gir E (2013). Fatores individuais, laborais e organizacionais associados à adesão às precauções-padrão. J Infect Control.

[15] Fukuda H, Imanakaa Y, Hirose M, Hayashida K (2009). Factors associated with system-level activities for patient safety and infection control. Health Policy.

[16] Landis J, Koch G (1977). The measurement of observer agreement for categorical data. Biometrics.

[17] Luo Y, He GP, Zhou JW, Luo Y (2010). Factors impacting compliance with standard precautions in nursing, China. Int J Infect Dis.

[18] Marziale MH, Zapparoli AS, Felli VE, Anabuki MH (2010). Rede de Prevenção de Acidentes de Trabalho: uma estratégia de ensino a distância. Rev Bras Enferm.

[19] Marziale MHP, Rocha FLR, Robazzi MLCC, Cenzi CM, Santos HEC, Trovó MEM (2013). Influência organizacional na ocorrência de acidentes de trabalho com exposição a material biológico. Rev Lat Am Enfermagem.

[20] Mathai E, Allegranzi B, Kilpatrick C, Pitted D (2010). Prevention and control of health care-associated infections through improved hand hygiene. Indian J Med Microbiol.

[21] Ministério da Saúde (2002). Portaria nº 2.224, de 5 de dezembro de 2002. Dispõe sobre o sistema de classificação hospitalar do Sistema Único de Saúde. Diario Oficial Uniao.

[22] Motamed N, Babamahmood A, Khalilian M, Peykanheirati M, Mozari M (2006). Knowledge and practices of health care workers and medical students towards universal precaution in hospitals in Mazandaran Province. East Mediterr Health J.

[23] Pasquali L (2009). Psicometria. Rev Esc Enferm USP.

[24] Reichenheim ME, Moraes CL (2007). Operacionalização de adaptação transcultural de instrumentos de aferição usados em epidemiologia. Rev Saude Publica.

[25] Sapnas KG, Zeller RA (2002). Minimizing sample size when using exploratory fator analusis for measuremen. J Nurs Meas.

[26] Siegel JD, Rhinehart E, Jackson M, Chiarello L (2007). 2007 Guideline for isolation precautions: preventing transmission of infectious agents in healthcare settings.

[27] Swinscow TDV (1997). Statistics at square one.

[28] Terwee CB, Bot SDM, Boer MR, Windt DAWM, Konl DL, Dekker J (2007). Quality criteria were proposed for measurement properties of health status questionnaires. J Clin Epidemiol.

[29] Valim MD, Marziale MHP, Hayashida M, Richart-Martínez M (2014). Ocorrência de acidentes de trabalho com material biológico potencialmente contaminado em enfermeiros. Acta Paul Enferm.

[30] Valim MD, Marziale MHP (2013). Adaptação cultural do “Questionnaires for Knowledge and Compliance with Standard Precaution” para o português brasileiro. Rev Gaucha Enferm.

[31] Waltz C, Strickland OL, Lenz E (2010). Measurement in nursing and health research.

[32] World Health Organization (2009). WHO Guidelines on hand hygine in health care: first global patient safety challenge, clean care is safer care.

