# Supplementary material for: Validity and reliability of the Questionnaire for Compliance with Standard Precaution
Source: Rev Saude Publica. 2015 Dec 16;49:87. doi: 10.1590/S0034-8910.2015049005975 (PMC4687826; doi:10.1590/S0034-8910.2015049005975)
Supplement: Translation

# Translation: Validade e confiabilidade do Questionário de Adesão às Precauções-Padrão

**Authors:** Marília Duarte Valim, Maria Helena Palucci Marziale, Miyeko Hayashida, Fernanda Ludmilla Rossi Rocha, Jair Lício Ferreira Santos

**Affiliations:** I Departamento de Enfermagem. Centro Universitário da Fundação de Ensino Octávio Bastos. São João da Boa Vista, SP, Brasil; II Departamento de Enfermagem Fundamental. Escola de Enfermagem de Ribeirão Preto. Universidade de São Paulo. Ribeirão Preto, SP, Brasil; III Departamento de Enfermagem. Seção de Apoio Laboratorial. Escola de Enfermagem de Ribeirão Preto. Universidade de São Paulo. Ribeirão Preto, SP, Brasil; IV Departamento de Medicina Social. Faculdade de Medicina de Ribeirão Preto. Universidade de São Paulo. Ribeirão Preto, SP, Brasil

**Keywords:** Enfermeiras, Precauções Universais, Capacitação, Questionários, Traduções, Reprodutibilidade dos Testes, Validade dos Testes, Estudos de Validação

## Abstract

**OBJETIVO:**

: Avaliar a validade e a confiabilidade do Questionário de Adesão às Precauções-padrão em enfermeiros.

**MÉTODOS:**

: Estudo metodológico realizado com 121 enfermeiros de estabelecimentos de saúde do interior de São Paulo, 2012, representados por dois estabelecimentos de alta complexidade e três de média complexidade. A consistência interna foi calculada pelo alfa de Cronbach e a estabilidade pelo coeficiente de correlação intraclasse, por meio de teste-reteste. Foi realizada a validação de construto convergente, divergente e por grupos conhecidos. RESULTADOS: O questionário mostrou-se confiável (alfa de Cronbach: 0,80; coeficiente de correlação intraclasse: 0,97). Quanto à validade de construto convergente e divergente, foi verificada forte correlação entre a adesão às precauções-padrão e a percepção de clima de segurança e a menor percepção de obstáculos para segui-las (r = 0,614 e r = -0,537, respectivamente). Enfermeiros que receberam treinamento sobre as precauções-padrão e atuantes nos estabelecimentos de maior complexidade mostraram-se mais aderentes (p = 0,028 e p = 0,006, respectivamente).

**CONCLUSÕES:**

: A versão brasileira do questionário de adesão às precauções-padrão mostrou-se válida e confiável. Futuras investigações devem ser realizadas com amostras de enfermeiros mais representativas da realidade brasileira. A utilização do questionário pode auxiliar na proposição de medidas educativas frente às possíveis lacunas identificáveis, com enfoque na saúde do trabalhador e na segurança do paciente.

## INTRODUÇÃO

As precauções-padrão incluem higienização das mãos, uso de equipamentos de proteção individual (EPI), prevenção de acidentes com materiais perfurocortantes, medidas de limpeza e desinfecção do ambiente, etiqueta ao tossir e práticas seguras na realização de injeções.^23^ Quando corretamente implementadas, são a principal estratégia para proteger o trabalhador da exposição a material biológico e prevenir as infecções relacionadas à assistência em saúde.^6,17^

Mesmo com a criação e divulgação dos protocolos sobre as precauções-padrão nas instituições de saúde, os acidentes de trabalho com material biológico potencialmente contaminado são frequentes.^5,16^ As infecções relacionadas à assistência em saúde são o efeito adverso mais comum e mais estudado da hospitalização. São o principal problema de saúde pública nos países desenvolvidos e em desenvolvimento devido à morbidade, mortalidade, hospitalização prolongada e efeitos negativos ao paciente e ao bem-estar econômico da nação.^29^

A adesão às precauções-padrão é mundialmente inferior ao preconizado.^3,7,26^ Algumas variáveis são apontadas como impactantes na adesão às precauções-padrão, como: treinamento prévio sobre precauções-padrão;^2,14^ percepção de clima de segurança organizacional;^4^ percepção de obstáculos para seguir as precauções-padrão;^11^ autoeficácia percebida;^14^ complexidade dos estabelecimentos de saúde – uma vez que a adesão mostrou-se maior em estabelecimentos maiores e mais complexos^12,14^ –, e conhecimento sobre tais medidas.^19^

A adesão às medidas de precauções-padrão como estratégia primária para prevenir acidentes de trabalho e doenças profissionais ocorridas por contato com material biológico potencialmente contaminado é essencial e deve ser avaliada pelas instituições de saúde, considerando variáveis individuais e da própria organização de trabalho.

Apesar da necessidade de usar instrumentos válidos e confiáveis na avaliação de construtos de interesse,^28^ poucos instrumentos na literatura mundial se propõem a medir tal construto.^2,8,14,19,27^ O *Questionnaire for Compliance with Standard Precaution*,^14^adaptado para o português do Brasil,^27^ possui características psicométricas adequadas e abrange conceitos importantes da adesão às medidas de precauções-padrão, como higienização das mãos, práticas seguras no manuseio de material perfurocortante e uso de EPI – luvas de procedimentos, máscaras, óculos e aventais de proteção.

O *Questionnaire for Compliance with Standard Precaution, *construído e validado em amostra de 1.444 enfermeiras chinesas,^14^ possui características psicométricas adequadas. É composto por 20 questões em formato de escala tipo *Likert*, de zero a quatro pontos, com intervalo de pontuação de zero a 80 pontos. A cada resposta “sempre” são somados quatro pontos, e às respostas “nunca” nenhum ponto é somado. O único item reverso é o de número 20. O referido questionário foi feito com base no *Questionnaire of Practice of Standard Precaution*, desenvolvido por Askarian et al^2^ e nas diretrizes internacionais^23^ sobre as medidas de precauções-padrão.

Objetiva-se dar continuidade ao processo de adaptação do Questionário de Adesão às Precauções-padrão (QAPP),^27^ por meio da análise da equivalência de mensuração. Três enfoques principais são sugeridos: avaliação da estrutura dimensional do instrumento, incluindo adequação dos itens que o compõem; análise da confiabilidade das informações; e verificação da validade do instrumento.^21^

Adotou-se como referencial teórico-metodológico o processo de validação de instrumentos de medidas, explicado por Pasquali.^20^ Segundo o autor, a legitimação de instrumentos só acontece quando existe uma teoria prévia que fundamente a representação comportamental, constituindo uma hipótese dedutível desta teoria.

O objetivo deste estudo foi avaliar a validade e a confiabilidade do Questionário de Adesão às Precauções-padrão adaptado para enfermeiros.

## MÉTODOS

Estudo metodológico, realizado com enfermeiros atuantes em instituições de saúde do interior de São Paulo, Brasil. Foi sorteada amostra aleatória de enfermeiros pertencentes a estabelecimentos de alta complexidade de um dos municípios do interior do Estado de São Paulo e enfermeiros pertencentes a estabelecimentos de média complexidade, de outro município do interior do mesmo estado. O critério de eleição de diferentes instituições, segundo o nível de complexidade, foi adotado para que fosse possível a realização do processo de validação de construto por grupos conhecidos ou grupos contrastantes.

Os estabelecimentos de alta complexidade foram representados por um hospital de ensino e uma unidade de emergência vinculada a esse hospital, classificados como porte IV.^18^ Os estabelecimentos de saúde de média complexidade estavam representados por um hospital privado filantrópico, um hospital privado e uma unidade de pronto atendimento, vinculada a um serviço privado de saúde.

Segundo Sapnas e Zeller,^22^ amostras de, no mínimo, 50 e, no máximo, 100 sujeitos são suficientes quando se pretende avaliar as propriedades psicométricas de instrumentos de medidas de construtos. Os autores acreditam que 10 respondentes por item represente uma amostra acima do necessário (*sample size overkill*).

O número de enfermeiros vinculados à unidade de emergência (125) e ao hospital de ensino (286) em 2011 totalizou 411 enfermeiros, segundo informações obtidas pelo setor de recursos humanos. O número de enfermeiros atuantes nos três estabelecimentos de saúde de média complexidade era de 39 enfermeiros. De acordo com Sapnas e Zeller,^22^ e a possibilidade de perdas e recusas, foi sorteada amostra de 120 enfermeiros pelo *software Statistical Package for Social Science* (SPSS) versão 16.0, vinculados ao hospital de ensino de alta complexidade. Optou-se por incluir os 39 enfermeiros dos serviços de média complexidade.

Foram incluídos enfermeiros que não estivessem em gozo de férias, licença saúde ou maternidade ou afastamento e que possuíssem mais de seis meses de experiência profissional. Foram excluídos os que exerciam exclusivamente atividades administrativas ou que estivessem participando de algum treinamento quando da realização da coleta de dados.

Os questionários autopreenchidos foram deixados em uma caixa lacrada posicionada na sala da chefia de enfermagem de cada setor, por uma semana, para posterior recolhimento. Esse procedimento garante maior anonimato ao participante, resultando em dados mais representativos da realidade. Nos setores onde não foi possível posicionar a caixa para coleta, foi solicitado aos enfermeiros que permanecessem com os questionários para posterior recolhimento pela pesquisadora, que o fazia no próximo plantão do trabalhador, por meio de três tentativas consecutivas.

A coleta de dados ocorreu de setembro a dezembro de 2012. Os questionários utilizados foram o sociodemográfico, a Escala de Clima de Segurança e a Escala de Percepção de Obstáculos para seguir as precauções-padrão, adaptadas e validadas para uso no Brasil,^4^ e o referido QAPP.^27^

Estabeleceu-se a hipótese de correlação convergente (positiva) entre a medida total do QAPP e a medida de percepção de clima de segurança organizacional para a validade de construto convergente. Para a validade de construto divergente, a hipótese foi de que há correlação negativa entre a medida do QAPP e a maior percepção de obstáculos para segui-las.

A Escala de Clima de Segurança, do tipo *Likert*, obteve alfa de Cronbach de 0,80 para o componente “ações gerenciais de apoio à segurança” e 0,69 para o componente “*feedback* das práticas seguras”. A Escala de Percepção de Obstáculos para seguir as precauções-padrão, também do tipo *Likert*, obteve alfa de Cronbach de 0,69. As escalas compõem um modelo explicativo de adesão às precauções-padrão, que engloba dimensões individuais, organizacionais e relacionadas ao trabalho.^4^

Estabeleceu-se a hipótese de que enfermeiros que receberam treinamento sobre as precauções-padrão eram mais aderentes a essas medidas do que os que afirmaram não ter recebido treinamento para a verificação da validade por grupos conhecidos. A validade por grupos conhecidos do QAPP testou a hipótese de que enfermeiros vinculados às unidades de alta complexidade aderiam mais às precauções-padrão do que enfermeiros atuantes em unidades de média complexidade.

A validade de conteúdo foi testada pela presença do efeito máximo e mínimo. A confiabilidade foi testada pelo teste-reteste, que representa a capacidade de um teste, medindo os mesmos sujeitos em ocasiões diferentes, ser capaz de produzir resultados idênticos, i.e., a correlação entre as duas medidas obtidas em diferentes momentos deve ser o mais próximo possível de 1.^20^ Para a realização do reteste, foi selecionada amostra de 30 enfermeiros, pertencentes aos estabelecimentos de alta complexidade, segundo a interpolação dos valores indicados na literatura,^9^ cujos resultados apontam para amostra de 28 indivíduos.

A validade de construto convergente e divergente foi realizada pela correlação de Pearson com a Escala de Clima de Segurança e a Escala de Percepção de Obstáculos para seguir as precauções-padrão. Valores menores que 0,30 correspondem a correlações fracas e de pouca aplicabilidade clínica; valores entre 0,30 e 0,50 são consideradas correlações moderadas e, acima de 0,50, correlações fortes.^1^ Para análise da validade por grupos conhecidos, foi utilizado o teste* t *de Student. Os referidos testes foram aplicados após a verificação da normalidade pelo teste de Shapiro-Wilk.

A consistência interna foi testada pelo cálculo do alfa de Cronbach, cujos valores entre 0,70 a 0,90 foram considerados aceitáveis.^10^ O coeficiente de correlação intraclasse (ICC) foi calculado para análise da estabilidade, por comparação entre a pontuação obtida pela aplicação do questionário no teste-reteste. Para a aplicação do reteste, foi respeitado o período de sete a 14 dias, conforme preconiza a literatura, com intervalo de 95% de confiança (IC95%).^25^

Buscou-se aferir um ICC de pelo menos 0,65 com amplitude de 0,3 no intervalo de confiança, com nível de significância de 5%. O valor de 0,65 para o ICC seguiu a indicação de Landis e Koch^13^ para uma concordância substancial.

Foi adotado nível de significância de 0,05 para todos os testes de hipóteses. Os testes compreenderam a significância ou não das correlações, pela técnica usual da correlação de Pearson e pela ANOVA com medidas repetidas para o ICC.

Os dados foram processados e analisados pelo SPSS, versão 16.0, para Windows 7.0. Para a compilação dos dados foi utilizada a técnica de dupla digitação para minimizar potenciais erros de digitação. Pesquisa aprovada pelo Comitê de Ética e Pesquisa da Escola de Enfermagem de Ribeirão Preto da Universidade de São Paulo (Protocolo 1.306/2011). Foram seguidas as normatizações da Resolução 196/96 do Conselho Nacional de Saúde sobre pesquisa envolvendo seres humanos. Todos os participantes assinaram termo de consentimento livre e esclarecido.

Após computadas as perdas e recusas, a amostra final foi de 121 enfermeiros, sendo 91 pertencentes às unidades de alta complexidade e 30, às de média complexidade. O percentual de resposta foi de 75,8% para as unidades de alta complexidade e 77,0% para os demais estabelecimentos de saúde. Quanto aos sujeitos incluídos para participarem do reteste, dos 30 sujeitos selecionados, dois se recusaram a realizá-lo e 28 compuseram a amostra final.

## RESULTADOS

A grande maioria (90,9%) dos profissionais era do sexo feminino. Dos enfermeiros das unidades de alta complexidade, 48,8% pertenciam ao hospital de ensino e 26,4%, à unidade de emergência vinculada a este hospital. Um total de 13,2% dos profissionais das unidades de média complexidade pertencia à santa casa filantrópica e 11,6% pertencia ao hospital particular e à unidade de pronto atendimento.

A média de idade dos trabalhadores foi de 36,2 anos (DP = 8,95), mediana de 34, idade máxima de 58 e mínima de 23 anos. A média do tempo de atuação profissional foi de 10,16 anos (DP = 7,22), mediana de oito anos. O maior tempo de atuação profissional foi representado por 30 anos de serviço e o tempo mínimo, por três meses.

Na análise descritiva do QAPP, foi obtida média de adesão de 62,2 pontos (DP = 8,47) e mediana de 63,0. O escore mínimo obtido foi 34 e o máximo 78 pontos (Tabela 1).

**Tabela 1:** Estatística descritiva dos valores obtidos pelo questionário de adesão às medidas de precauções-padrão pelos enfermeiros (n = 121). Serviços de saúde de municípios do interior do Estado de São Paulo, Brasil, 2012.

Variável	Sempre (4)		Frequentemente (3)		Às vezes (2)		Raramente (1)		Nunca (0)	

	f	%	f	%	f	%	f	%	f	%
1. Realiza a higiene das mãos no intervalo entre a prestação de cuidados a diferentes pacientes.	73	60,3	45	37,2	3	2,5	–	–	–	–
2. Realiza a higiene das mãos após retirar as luvas.	89	73,5	29	24,0	3	2,5	–	–	–	–
3. Realiza a lavagem das mãos imediatamente após contato com materiais biológicos potencialmente contaminados.	111	91,7	10	8,3	–	–	–	–	–	–
Relato da frequência do uso das luvas nos procedimentos em que há possibilidade de contato com materiais biológicos potencialmente contaminados, listados abaixo.										
4. Coleta de sangue.	63	52,1	33	27,3	17	14,0	8	6,6	–	–
5. Procedimentos que envolvam a possibilidade de contato com urina ou fezes.	96	79,3	20	16,5	3	2,5	2	1,7	–	–
6. Procedimentos que envolvam a possibilidade de contato com a pele não íntegra do paciente.	89	73,6	20	16,5	9	7,4	3	2,5	–	–
7. Procedimentos que envolvam a possibilidade de contato com a mucosa do paciente.	91	75,2	22	18,2	5	4,1	3	2,5	–	–
8. Procedimentos que envolvam a possibilidade de contato com secreções das vias aéreas do paciente.	109	90,0	7	5,8	2	1,7	2	1,7	1	0,8
9. Injeção intramuscular ou subcutânea.	16	13,2	38	31,4	31	26,5	22	18,2	14	11,6
10. Realização de curativos.	83	68,6	21	17,4	16	13,2	1	0,8	–	–
11. Limpeza para a remoção de sangue.	105	86,8	11	9,1	5	4,1	–	–	–	–
12. Punção venosa.	50	41,3	34	28,1	30	24,8	7	5,8	–	–
13. Contato com amostras de sangue.	48	39,7	47	38,8	14	11,6	9	7,4	3	2,5
14. Utiliza máscara de proteção quando há possibilidade de contato com respingo de sangue, fluido corporal, secreção ou excreção.	20	16,5	69	57,0	23	19,0	7	5,8	2	1,7
15. Utiliza óculos de proteção quando há possibilidade de contato com respingo de sangue, fluido corporal, secreção ou excreção.	15	12,4	44	36,4	43	35,5	16	13,2	3	2,5
16. Utiliza avental de proteção quando há possibilidade de contato com respingo com sangue, fluido corporal, secreção ou excreção.	15	12,4	35	28,9	24	19,8	30	27,9	17	14,0
17. Utiliza gorros e propés descartáveis quando há possibilidade de contato com respingos de sangue, fluido corporal, secreção ou excreção.	9	7,5	17	14,0	26	21,5	30	24,8	39	32,2
18. Não realiza o encape ativo de agulhas usadas ou realizo o encape passivo de agulhas apenas com uma mão.	53	43,8	23	19,0	27	22,3	10	8,3	8	6,6
19. Descarta as agulhas, lâminas e outros materiais perfurocortantes em recipientes de descarte específicos.	119	98,3	2	1,7	–	–	–	–	–	–
20. Após acidentes de trabalho com materiais perfurocortantes potencialmente contaminados, espreme imediatamente o local, em seguida efetua a antissepsia e coloca um curativo.	41	33,8	11	9,1	5	4,1	10	8,3	48	36,7

Embora 91,7% dos enfermeiros tenham relatado que sempre realizavam a lavagem das mãos após contato com material biológico potencialmente contaminado, 39,7% do total de enfermeiros da amostra não as higienizavam no intervalo entre o cuidado a diferentes pacientes. Cerca de 52,1% dos enfermeiros sempre utilizavam luvas na coleta de sangue, 13,2% na realização de injeção intramuscular ou subcutânea, 68,6% na realização de curativos e 41,3% na punção venosa. Quanto às máscaras de proteção, 16,5% dos enfermeiros as usavam quando havia possibilidade de contato com material biológico potencialmente contaminado; 35,5% utilizavam óculos de proteção às vezes e 15,7%, raramente ou nunca. Um total de 19,8% raramente ou nunca utilizava os aventais de proteção.

Apenas os itens 9, 15 e 16 não sofreram influência do efeito máximo e mínimo (Tabela 1). A existência dos efeitos máximo e mínimo foi observada quando mais de 15,0% dos participantes optavam pelo menor ou pelo maior escore possível na escala de resposta, respectivamente.

O coeficiente alfa de Cronbach total foi de 0,80 (Tabela 2).

**Tabela 2:** Coeficiente de correlação item-total, valor do alfa do total dos 20 itens do questionário de adesão às precauções-padrão e valores de alfa quando cada um dos itens foi excluído. Serviços de saúde de municípios do interior do Estado de São Paulo, Brasil, 2012

Itens do questionário de adesão às precauções-padrão	Média, se item removido	Variância, se item removido	Correlação item-total corrigida	Alfa de Cronbach, se item removido
1. Realiza a higiene das mãos no intervalo entre a prestação de cuidados a diferentes pacientes.	78,05	65,62	0,42	0,78
2. Realiza a higiene das mãos após retirar as luvas.	77,91	68,16	0,14	0,79
3. Realiza a lavagem das mãos imediatamente após contato com materiais biológicos potencialmente contaminados.	77,71	68,52	0,25	0,79
Relate a frequência do uso das luvas nos procedimentos em que há possibilidade de contato com materiais biológicos potencialmente contaminados, listados abaixo.				
4. Realiza coleta de sangue.	78,39	59,73	0,63	0,76
5. Executa procedimentos que envolvam a possibilidade de contato com urina ou fezes.	77,90	64,32	0,52	0,78
6. Executa Procedimentos que envolvam a possibilidade de contato com a pele não íntegra do paciente.	78,02	63,94	0,46	0,78
7. Executa procedimentos que envolvam a possibilidade de contato com a mucosa do paciente.	77,98	63,64	0,50	0,78
8. Executa procedimentos que envolvam a possibilidade de contato com secreções das vias aéreas do paciente.	77,81	64,36	0,49	0,78
9. Aplica injeção intramuscular ou subcutânea.	79,49	57,35	0,59	0,76
10. Realiza curativos.	78,11	65,54	0,28	0,79
11.Faz Limpeza para a remoção de sangue.	77,81	65,68	0,48	0,78
12. Executa punção venosa.	78,61	58,97	0,68	0,76
13. Tem contato com amostras de sangue.	78,58	60,22	0,53	0,77
14. Utiliza máscara de proteção quando há possibilidade de contato com respingo de sangue, fluido corporal, secreção ou excreção.	78,81	63,19	0,44	0,78
15. Utiliza óculos de proteção quando há possibilidade de contato com respingo de sangue, fluido corporal, secreção ou excreção.	79,22	59,97	0,58	0,77
16. Utiliza avental de proteção quando há possibilidade de contato com respingo com sangue, fluido corporal, secreção ou excreção.	79,61	59,31	0,45	0,78
17. Utiliza gorros e propés descartáveis quando há possibilidade de contato com respingos de sangue, fluido corporal, secreção ou excreção.	80,22	61,20	0,34	0,78
18. Não realiza o encape ativo de agulhas usadas ou realizo o encape passivo de agulhas apenas com uma mão.	78,82	65,23	0,13	0,80
19. Descarta as agulhas, lâminas e outros materiais perfurocortantes em recipientes de descarte específicos.	77,65	69,38	0,16	0,79
20. Após acidentes do trabalho com materiais perfurocortantes contaminados, espreme imediatamente o local, em seguida lavo, efetuo a antissepsia e coloco um curativo.	79,63	66,39	-0,001	0,83

O ICC obtido pelo teste-reteste do QAPP foi de 0,973 (IC95% 0,929;0,987), o que significa uma classificação quase perfeita.

Quanto à validade do QAPP, foi encontrada forte correlação (r = 0,614 e p ≤ 0,001) entre a adesão às precauções-padrão e maior percepção de clima de segurança pelos enfermeiros. Com relação à validade de construto divergente, foi verificada correlação forte (r = -0,537 e p ≤ 0,001) entre a adesão às precauções-padrão e menor percepção de obstáculos para seguir as precauções-padrão.

Do total de enfermeiros incluídos na presente amostra, 15 relataram não ter recebido treinamento sobre as medidas de precauções-padrão. O grupo de enfermeiros que recebeu treinamento apresentou média de escore no QAPP de 66,8 (n = 15) e o grupo que não recebeu treinamento obteve média de 62,8 (n = 98), com diferenças estatisticamente significantes (p = 0,028).

A adesão às precauções-padrão diferiu significativamente entre os grupos (p = 0,006). O grupo de enfermeiros pertencentes a estabelecimentos de alta complexidade (n = 87) obteve média de adesão de 68 e o grupo pertencente às demais instituições de saúde (n = 28) obteve 63,2.

## DISCUSSÃO

Instrumentos devem ser válidos e confiáveis quando da proposição da aferição de determinado construto. A viabilidade de um instrumento fidedigno para aferir a adesão de enfermeiros às precauções-padrão é importante para o contexto brasileiro da saúde ocupacional e segurança do paciente.

A análise descritiva do instrumento mostrou a presença de efeito máximo e mínimo no instrumento. Apenas os itens 9, 15 e 16 não sofreram influência desses efeitos. Isso pode levar a repercussões negativas quanto à validade de conteúdo do instrumento, o que pode indicar representatividade subótima do construto investigado pelos itens do instrumento.^28^ Uma possível justificativa quanto à presença do efeito máximo é a de que estudos observam autorrelatos de adesão com índices superiores aos realmente constatados por estudo observacional,^3,15^ uma vez que profissionais tendem a superestimar aquilo que realmente executam na prática.

Com relação à confiabilidade, foi observada consistência interna desejável, descrita como a capacidade de verificar a correlação (homogeneidade) entre os itens de um instrumento com formato de escala, i.e., verificar se medem o mesmo construto teórico a que se propõem. A medida de consistência interna obtida pelo alfa de Cronbach é importante e desejável quando se trabalha com instrumentos que objetivam medir construto único por meio de múltiplos itens. O valor deve variar de 0,70 a 0,95.^10^

Ainda com relação à consistência interna, os itens 2, 18 e 20 apresentaram baixa correlação com a dimensão pertencente, o que pode sugerir dimensão diferente da verificada no instrumento. No entanto, nenhum desses itens alteraria, se retirado, o valor de alpha em mais do que 5,0%, motivo pelo qual optou-se por mantê-los, até que futuros estudos indiquem o contrário. O item 20 foi o único não respondido por seis enfermeiros e, se excluído, acarretaria em um valor de alfa total de 0,83. O item foi mantido em função de sua relevância. Não foi realizada verificação por componentes principais das eventuais dimensões estruturais do instrumento. O alto valor de sua consistência interna indica a unidimensionalidade ou, pelo menos, a proeminência de uma dimensão.

A estabilidade mostrou-se satisfatória, o que confirma que a ferramenta pode ser aplicada em diversos momentos a fim de que possíveis alterações possam ser detectadas na amostra estudada.

A validade de construto tem o objetivo de sustentar a habilidade do instrumento para medir o que se designa a medir. A lacuna desse tipo de validade pode afetar negativamente os resultados obtidos pela aplicação de um instrumento.^28^A forte correlação evidenciada pelos testes de validade entre adesão às precauções-padrão e maior percepção de clima de segurança organizacional está de acordo com achados científicos.^4,11^Isso reforça a hipótese de que o instrumento realmente mede o construto que se propõe a avaliar. No entanto, os valores de correlação sofrem classificações ligeiramente diferentes segundo estudos,^24^ nos quais valores entre 0,4 e 0,6 são correlações moderadas e valores acima de 0,7 são consideradas fortes.

Instituições de saúde com culturas de segurança fortes registram menores números de acidentes de trabalho quando comparadas às instituições com culturas mais fracas. A menor ocorrência não se deve somente à presença de programas de segurança efetivos e bem desenvolvidos nos estabelecimentos, mas também à gestão da instituição por meio desses programas, indicando sinais de comprometimento com a segurança de seus trabalhadores.^7^

A validade por grupos conhecidos do QAPP fortalece o questionário. O treinamento e a educação permanente dos profissionais de saúde são variáveis que aumentam a adesão às medidas de segurança, pois são medidas essenciais para garantir que políticas de prevenção e controle de infecções relacionadas à assistência em saúde e medidas de segurança ocupacional sejam compreendidas e aderidas.^3^

A validade por grupos conhecidos também se mostrou satisfatória com relação a enfermeiros de estabelecimentos de alta complexidade serem mais aderentes às precauções-padrão do que os atuantes em estabelecimentos de média complexidade. Recursos humanos e financeiros variam de forma significativa entre os tipos de estabelecimentos de saúde e, assim, hospitais de ensino e de maior complexidade tendem a exercer práticas de controle de infecção mais eficazes que hospitais municipais ou filantrópicos, menos complexos e que recebem menos incentivos financeiros.^12^

Os achados do estudo confirmam que o QAPP encontra-se adaptado e pode ser utilizado em enfermeiros no Brasil. Futuros achados podem fortalecer o instrumento ou evidenciar limitações em sua utilização.

Com relação à participação em treinamentos pelos enfermeiros, futuras pesquisas poderão investigar os conteúdos abordados, os métodos utilizados e a periodicidade dos treinamentos realizados. Dessa forma, seria possível obter análise descritiva mais fundamentada acerca dos treinamentos oferecidos. Isso pode auxiliar na verificação de variáveis com possibilidade de influência na adesão dos enfermeiros às precauções-padrão, além de colaborar com a validade do questionário e sustentar propostas intervencionistas.

A amostra do estudo é pouco representativa da realidade de enfermeiros brasileiros. Outros estudos são necessários, com maior número de sujeitos e amostras mais heterogêneas e menos localizadas. Investigações com amostras de enfermeiros atuantes em diversas instituições de saúde de diferentes regiões do País são recomendadas para avaliar interferências regionais. Deve-se considerar o regionalismo e a grande extensão territorial do Brasil, bem como as características sociais, econômicas e culturais peculiares às instituições de saúde.

O questionário de adesão às precauções-padrão apresentou confiabilidade satisfatória, calculada pela consistência interna e estabilidade. O referido questionário respondeu satisfatoriamente aos testes para validade de construto divergente, convergente e por grupos distintos. Futuras investigações deverão prosseguir com o processo de verificação da equivalência de mensuração do questionário por meio da análise da validade dimensional dos componentes principais do instrumento.
